# Comprehensive Investigation of the Effects of Brewing Conditions in Sample Preparation of Green Tea Infusions

**DOI:** 10.3390/molecules24091735

**Published:** 2019-05-04

**Authors:** Yan Jin, Jing Zhao, Eun Mi Kim, Ki Hyun Kim, Seulgi Kang, Heesoo Lee, Jeongmi Lee

**Affiliations:** 1 School of Pharmacy, Sungkyunkwan University, Suwon 16419, Korea; kimyeon909@gmail.com (Y.J.); em131632@gmail.com (E.M.K.); khkim83@skku.edu (K.H.K.); seulgi3262@naver.com (S.K.); yeeheesoo@naver.com (H.L.); 2 School of Pharmacy, Shenyang Pharmaceutical University, Shenyang 110016, China; jingzhao0225@sina.com

**Keywords:** green tea, brewing conditions, antioxidant activity, catechins, metabolomics

## Abstract

Chemical and biological investigation of green tea has been generally performed while using different infusions that are prepared without consideration of the effects of sample preparation conditions. In this study, for the first time, the effects of green tea brewing conditions on the antioxidant activity and chemical profiles of metabolome and catechin compounds were examined at 60 °C and 95 °C for a period of 5–300 min. The antioxidant capacities of the tea infusions, which were assessed as per 2,2-diphenyl-1-picryl-hydrazyl hydrate (DPPH) radical scavenging activity, depended more on temperature than time. Metabolomics study that was based on ultra-high performance liquid chromatography-quadrupole-time-of-flight mass spectrometry (UHPLC-QTOF/MS) revealed that the metabolic profiles, including 33 differential metabolites, were significantly changed by temperature and time, with the effects of time being more evident at 95 °C starting after 30 min. Infusions that were brewed at 95 °C for greater than 30 min yielded distinct profiles in the hierarchical clustering analysis. The quantification of eight catechins by UHPLC-QqQ/MS showed that the total catechin level peaked at 95 °C brewing at 10 min, after which the levels of four epi-forms of catechins decreased and those of four non-epi-forms increased, implying the epimerization of catechins over time. These results suggest that the brewing conditions for sample preparation of green tea should be put into careful consideration in studies where green tea extracts are applied as aqueous infusions.

## 1. Introduction

Green tea, *Camellia sinensis*, is being increasingly consumed, not only for its unique taste and flavor, but also its prospective health promoting effects [1]. Numerous beneficial activities of green tea have been reported, including antimicrobial, hypolipidemic, hepatoprotective, and bone-improving effects [2]. Green tea is a type of unfermented tea that contains a variety of bioactive compounds, such as amino acids, vitamins, phenolic acids, flavonols, and catechins [3]. In particular, catechins have been of great interest, because they have anti-oxidant [4], anti-cancer [5], anti-bacterial [5], anti-diabetic [6], and anti-angiogenesis activities [7]. Catechins can be converted to more complex and oxidized species, such as theaflavins and thearubigins, during fermentation [8]; therefore, catechins are present in larger amounts in green tea than any other types of fermented tea and they are considered to be the most valuable and unique components in green tea [9].

Factors, such as harvest season [10], origin [11], and manufacturing process [12], can affect the composition and content of the bioactive compounds in tea leaves. With regard to tea infusions, the sensory properties and color of tea change with different brewing conditions [13], and brewing temperature and time are considered as the most impactful factors for variation [14,15]. For example, extended thermal treatment can render infusions dark and bitter [16]. The antioxidant activities, total phenolic contents, and total flavonoid contents varied depending on the brewing temperature and length of hot water infusion, and the primary components of green tea, namely, gallic acid, caffeine, catechins, and flavonols, also changed depending on the brewing conditions [17,18,19]. Among various compounds, catechins can be destroyed by brewing above 90 °C [20] and can also undergo degradation, epimerization, and oligomerization at higher temperatures and longer brewing times [21,22].

Nonetheless, chemical and biological investigation of tea has been still performed without any consideration of the effects of sample preparation conditions. Different extraction solvents have been applied to examine the bioactivities and chemical profiles of green tea. For example, water [23], 70% methanol [24], 100% methanol [23,25], and solvent mixtures [26] were used to prepare green tea extracts in metabolomics investigation, and the ethanolic extract was administered to a mouse model to examine the biological effects of green tea [27]. Besides diverse extraction solvents, even the aqueous infusions or extracts of green tea have been prepared under numerous brewing conditions [23,25], sometimes with unspecified extraction conditions [23]. Likewise, sample preparation procedures were all different and not carefully selected for other types of tea infusions, such as Pu-erh tea [28,29].

In this study, we aimed to comprehensively investigate the effects of brewing conditions on the chemical profiles and bioactivity of green tea infusions. To this end, we first assessed the antioxidant activity of various tea infusions that were brewed at low (60 °C) and high (95 °C) temperatures for a relatively long period (5–300 min). Subsequently, a metabolic profiling with appropriate multivariate statistical analyses was conducted based on ultra-high performance liquid chromatography-quadrupole-time-of-flight mass spectrometry (UHPLC-QTOF/MS). Finally, a quantitative analysis of major target compounds comprising eight catechins was conducted while using UHPLC-QqQ/MS. This study provides the evidence that samples of green tea infusions should be carefully prepared in consideration of the effects of brewing conditions on the metabolic profiles and activity.

## 2. Results and Discussion

### 2.1. Effects of Brewing Conditions on Antioxidant Activity

**Figure 1:**
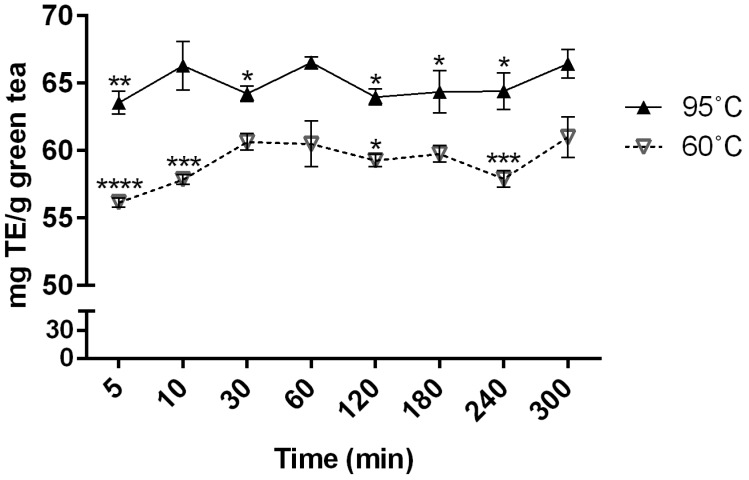
DPPH free radical scavenging activity of green tea infusions brewed at 60 °C and 95 °C for 5–300 min. Each time point was compared with 300 min and 60 min for 60 °C and 95 °C, respectively. *, **, ***, and **** indicate *p* < 0.05, *p* < 0.01, *p* < 0.001, and *p* < 0.0001, respectively. All comparisons between 60 °C and 95 °C at the same time point were statistically significant (*p* < 0.001 for 5 min and 120 min; and, *p* < 0.01 for 10 min, 30 min, 60 min, 180 min, 240 min, and 300 min).

Changes in the antioxidant capacity of green tea infusions were monitored at low (60 °C) and high (95 °C) brewing temperatures for 5–300 min. The two selected temperatures could represent very different, yet practical, temperatures for tea brewing, so that the effects of brewing temperature could be revealed. 2,2-Diphenyl-1-picryl-hydrazyl hydrate (DPPH) assay, which is one of the most common antioxidant assays, was used to measure radical scavenging activity of the infusions [30]. Our results showed that the antioxidant activities of the different infusions were more dependent on brewing temperature than the brewing time (Figure 1). The antioxidant capacity at 95 °C was significantly higher than at 60 °C, regardless of brewing time (*p* < 0.05). While antioxidant capacity at 95 °C did not greatly vary among time points, the capacity at 60 °C tended to increase with time, and the antioxidant activity at 300 min. was significantly higher than those at shorter times among 60 °C infusions (*p* < 0.05). These results were similar to previous studies [18,19], although the monitoring period (5 h) of the present study was much longer than those of most studies (<1 h).

### 2.2. Effects of Brewing Conditions on Metabolic Profiles—Investigation via PCA and OPLS-DA Studies

Non-targeted UHPLC-QTOF/MS analysis was performed to understand the effects of brewing conditions on metabolic changes of tea infusions. The % RSD values of the retention time and peak area of the two ISs in the Quality control (QC) samples were below 0.2% and 6.5%, respectively, ensuring the stable status of the analytical instruments during analysis. In three-dimensional principal component analysis (PCA) score plots of both positive (POS) and negative (NEG) modes (Figure S1), the samples from 60 °C and 95 °C brewing were separately clustered, and the QC samples were also well separated from the two groups. The samples obtained from brewing at 95 °C exhibited a tendency to scatter from the 60 °C samples as a function of time while the 60 °C infusions were generally mingled with no noticeable time-dependent trend. These implied that compositions of green tea infusions changed according to brewing conditions and that the variations were more distinct at 95 °C than 60 °C.

In each group of samples brewed at the same temperature, a pairwise comparison was conducted relative to the 5 min. sample using orthogonal projections to latent structures-discriminant analysis (OPLS-DA). Samples from 60 °C and 95 °C at the same brewing time were also compared. A total of 22 OPLS-DA models showing unequivocal classification were established, and they were all valid with good classification and predictive ability (data not shown). Variable importance in projection (VIP > 1.0) values of the models were used to select the differential variables in combination with *t*-test (*p* < 0.05). In this way, differential metabolites could be selected as a function of brewing time and temperature. In total, 684 and 149 variables were selected as the potential discriminant variables from POS and NEG modes, respectively, and the selected variables were confirmed on chromatograms and the original dataset to avoid false positive results. Among the selected variables, 33 marker candidates were identified, including sugars, organic acids, amino acids, dipeptides, phenolic acids, polyphenols, flavonoids, and catechins. They comprised 14 compounds that were identified using authentic standards and 19 compounds putatively identified by comparison with existing databases or literature (Table 1). Details regarding the putative identification of several compounds are described in Supplementary Materials.

### 2.3. Effects of Brewing Conditions on Metabolic Profiles—Investigation via HCA Studies

**Figure 2:**
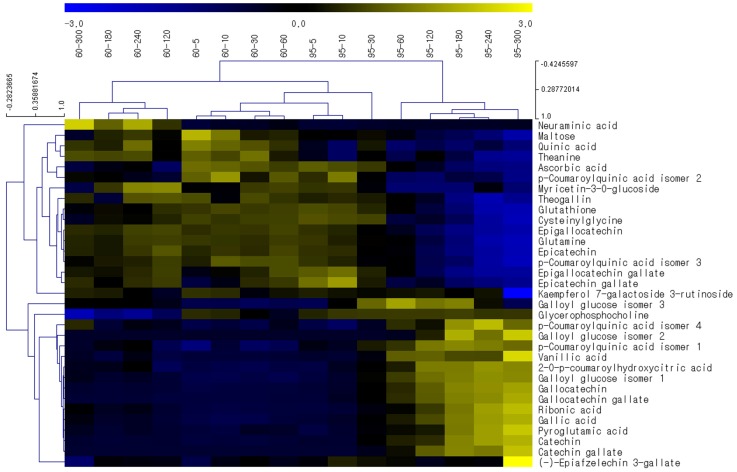
Hierarchical clustering and heat map of 33 differential marker compounds.

Hierarchical cluster analysis (HCA), which is an unsupervised multivariate technique, was conducted on the 33 differential metabolites in order to visualize the relationships among the 33 metabolites based on their relative levels across samples [31]. The results are displayed as dendrogram and heat map (Figure 2). Two main clusters of infusions were identified in the dendrogram: infusions brewed at 95 °C for 60–300 min. (right cluster) and the remaining infusions (left cluster), which is similar to findings in the PCA score plot (Figure S1). The heat map further revealed that these two clusters have contrasting patterns in the relative concentration levels of 33 metabolites and therefore that the green tea infusions brewed at 95 °C for 60–300 min. have very different metabolite profiles than the others. For selected compounds with high relative intensity and/or drastic changes, depending on brewing conditions, their changing trends were displayed for both brewing temperatures as a function of brewing time (Figure S2).

In Figure 2, the top half comprised 17 metabolites, including amino acids (glutamine, theanine, and cysteinylglycine) and epi-structured catehins (epicatechin, EC; epigallocatechin, EGC; epicatechin gallate, ECG; and epigallocatechin gallate, EGCG). Their levels were generally lower in the right cluster with a tendency to decrease with a longer brewing time (Figure S2). The opposite phenomenon was observed in the bottom half, which contained 16 metabolites, including non-epi-structured catechins (catechin, C; catechin gallate, CG; gallocatechin, GC; and gallocatechin gallate, GCG), phenolic acids (vanillic acid and gallic acid), and an amino acid (pyroglutamic acid). While their levels remained unaltered in the left cluster, they increased with time in the right cluster.

The levels of gallic acid and vanillic acid increased with increasing the brewing time and higher temperature (Figure S2). The production of gallic acid, which has strong antioxidant activity in tea infusions, is increased during fermentation and hydrolysis of galloylated catechins [9]. In contrast with gallic acid, the level of glutamine tended to decrease with higher temperatures and longer brewing times, as did that of theanine (Figure S2), which is the most abundant amino acid in tea. In previous studies [32,33], the levels of amino acids differently altered depending on the temperature and time. Although the theanine and glutamine levels slightly increased with time at high temperature, they were measured for up to 15 min, and the levels of some amino acids remained unchanged or even declined between 5–15 min [32]. Thus, the theanine and glutamine contents might have declined at longer times [32].

It is noteworthy that the metabolic profile results did not match with the antioxidant capacity results. The metabolic profiles changed massively among the time points, although the antioxidant capacity did not vary greatly or show any trends. For example, the antioxidant capacities at 95 °C were not significantly different between at 10 min and at 300 min (*p* = 0.9090; Figure 1); however, the profiles of 33 marker metabolites, many of which are bioactive compounds, presented totally different patterns between 10 min and 300 min at 95 °C brewing (Figure 2). Meanwhile, the concentrations of polyphenols, flavonoids, and catechins are known to be significantly correlated with the antioxidant activity of tea infusions [17]. Our results indicate that the apparent antioxidant capacities of infusions cannot represent the altering compositions and contents of various antioxidant compounds.

### 2.4. Effects of Brewing Conditions on Catechin Compositions

Among the differential markers, the most dramatic changes were observed in catechins, which are the most prevalent bioactive compounds in green tea. As shown in Figure 2, epi-forms (EC, ECG, EGC, and EGCG) remained largely unaltered at 60 °C; however, at 95 °C, they started to drastically decrease from 60 min, while the non-epi-forms (C, CG, GC, and GCG) dramatically increased in a time-dependent manner. More accurate determination of altering levels of the eight catechins was achieved using the UHPLC-QqQ/MS method under the experimental conditions in Section 3.4 (see Figure S3 for MRM chromatograms). The analytical method was validated in terms of linearity, precision, and accuracy, and the measured validation parameters were within acceptable ranges (Tables S1 and S2).

**Figure 3:**
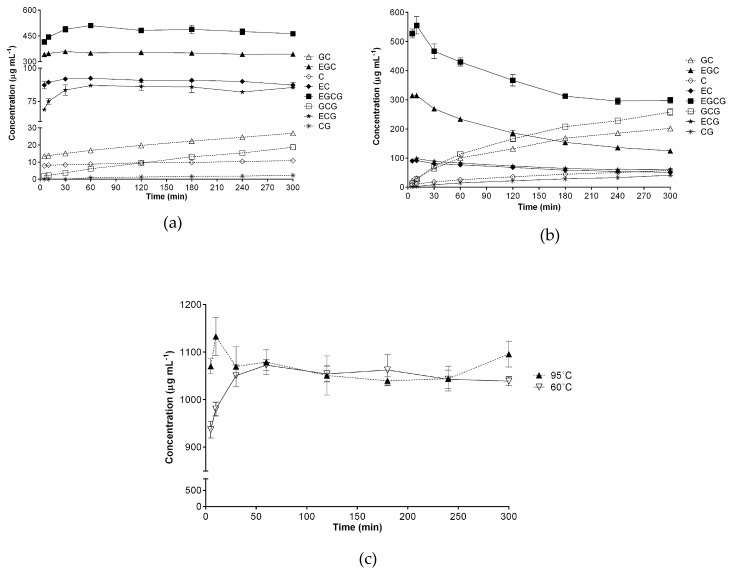
Quantification of eight catechins in green tea infusions brewed at 60 °C (**a**); 95 °C (**b**); and, total catechin concentrations (**c**).

Figure 3 displays the quantitative results. The concentrations of epi-structured catechins remained higher than those of non-epi forms at a brewing temperature of 60 °C. The levels of the four epicatechins reached a peak at between 30–60 min and then slightly decreased, while those of the four non-epicatechins gradually increased with time (Figure 3a). These results confirmed that epimerization from epi to non-epi forms slowly occurred at 60 °C. In contrast, 95 °C brewing gave rise to fairly different results than 60 °C (Figure 3b). The concentrations of the four epicatechins peaked at 10 min, after which they decreased drastically. In the meantime, levels of the non-epicatechins increased steadily for 300 min, with levels of some of the non-epi compounds (CGC and GC) surpassing those of EC and ECG after 60 min.

Brewing temperature and time are crucial factors in catechin extraction and epimerization [22,34], which was also confirmed in our study. The total catechin levels peaked at 95 °C for 10 min. (Figure 3c). This is consistent with the results of Perva-Uzunalić et al., who reported that the highest catechin extraction was also achieved at 80 °C for 20 min. [35]. Our brewing at 60 °C required a longer time, 60 min. to peak (Figure 3a) than 80 °C [35]. However, equal levels were observed at 60 °C and 95 °C after 60 min and they were generally unaltered, which we took as an indication of increased non-epicatechin content compensating for decreased epicatechins. The antioxidant capacity of green tea infusions was largely maintained for 5 h despite the decreased levels of more potent epi-structured catechins (Figure 1). Instead, the relative levels of other antioxidant phenolic compounds, such as gallic acid, were elevated (Figure S2). These observations confirm that the apparent antioxidant capacity of green tea infusions is a reflection of a wide variety of compounds. A number of studies have suggested catechins to be QC markers of green tea and its related products [36,37]. However, their levels in aqueous infusions are liable to change depending on the sample preparation conditions, as evidenced in this study. Accordingly, caution should be exercised in studies where green tea extracts are involved, such as in the assessment of the chemical or biological properties of green tea and the selection of QC markers for green tea-related products.

## 3. Materials and Methods

### 3.1. Chemicals and Reagents

Commercially available green tea leaves that are marketed under the brand name Osulloc were purchased from a domestic market (Seoul, Korea) and directly used for experiments. Green tea leaves were pulverized using an electric mill (Rong Tsong Precision Technology Co., Taichung, Taiwan) in order to ensure optimal and reproducible extraction [10,38]. The resulting powders were tightly sealed and stored at −40 °C until use after filtration through a 1 mm sieve.

Analytical standards of (−)-EGCG (>98%), (−)-GCG (>98%), (−)-EC (>98%), (−)-C (>98%), (−)-EGC (>98%), (+)-GC (>98%), (−)-ECG (>98%), and (−)- CG (>98%) were obtained from Biopurify Phytochemicals Ltd. (Chengdu, China). DPPH, (±)-6-hydroxy-2,5,7,8-tetramethylchromane -2-carboxylic acid (Trolox; 97%), chlorpropamide (≥97%), 4-chloro-dl-phenylalanine (≥97%), formic acid (FA, ≥98%), glutamine (≥99%), maltose (≥99%), and glycerophosphorylcholine (≥98%) were purchased from Sigma-Aldrich (St. Louis, MO, USA). HPLC-grade water, methanol (MeOH), and acetonitrile (ACN) were purchased from Honeywell Burdick & Jackson (Ulsan, Korea).

### 3.2. Preparation of Tea Infusions

Tea infusions were prepared by brewing 2.0 g of green tea powder in 200 mL doubly distilled water in a 250 mL Duran bottle. The water was pre-heated to the designated temperature prior to brewing and the bottle was protected from light during brewing in a water bath maintained at 60 °C or 95 °C, in order to suppress other variations than the brewing temperature and time. The solid-to-liquid ratio was according to the ordinary green tea brewing directions (e.g., 2–3 g per 6–8 ounces). The infusions were briefly stirred at specified intervals (5, 10, 30, 60, 120, 180, 240, and 300 min), at which time a 1 mL aliquot was transferred to an ice bath to quench the infusion. After thorough cooling and centrifugation, the cleared supernatant was diluted 10-fold with 50% MeOH and then passed through a 0.2 µm membrane filter from Millipore (Tullagreen, Ireland) prior to injection. Diluted infusions were spiked with chlorpropamide and 4-chloro-dl-phenylalanine as internal standards (IS) at 100 μg mL^−1^ for non-targeted analysis using UHPLC-QTOF/MS. QC samples were prepared by mixing an equal volume of all samples and were analyzed at an interval of every eight samples in the running sequence to monitor system stability. In contrast, the samples for targeted analysis of catechins using UHPLC-QqQ/MS contained no IS.

### 3.3. Antioxidant Assay

The antioxidant capacity of green tea infusions was evaluated by DPPH free radical scavenging assay, as previously described [39]. In brief, 100 µL of 300-fold diluted infusions in 50% MeOH was mixed with an equal volume of freshly prepared 0.2 mM DPPH in MeOH in a 96-well plate. After a 30 min. incubation period in the dark at room temperature, absorbance was measured at 517 nm to calculate scavenging activity, which was expressed as Trolox equivalents (mg TE/g green tea). The linear calibration range for Trolox equivalents was 10–100 µg mL^−1^ (y = 0.8032x − 0.5489, r^2^ = 0.9992). All of the measurements were conducted in triplicates.

### 3.4. Instruments and Operation Conditions

Non-targeted analysis was performed with an Acquity UPLC^TM^ system (Waters Co., Milford, MA, USA) coupled to a Waters Acquity Xevo G2 Q-TOF system (Waters Corp., Manchester, UK), as previously described by our group [40]. For each sample, 5 µL was loaded onto an Agilent ZORBAX Eclipse Plus C_18_ column (100 mm × 2.1 mm, 1.8 µm), and a gradient elution of the mobile phase consisting of 0.3% FA in water (A) and 0.3% FA in ACN (B) was performed, as follows: 0–1 min, 5–10% B; 1–10 min, 10–18% B; 10–14 min, 18–100% B; and, 14–17 min, 100% B. The flow rate and column temperature were set at 0.3 mL min^−1^ and 30 °C, respectively. The MS system was equipped with an electrospray ionization (ESI) source and was operated in both negative (NEG) and positive (POS) ionization modes with a data acquisition range of 100 to 1500 *m*/*z*. The conditions for the ESI source were optimized, as follows: capillary voltage, 3.0 kV (POS)/1.8 kV (NEG); sample cone, 30 V (POS)/45 V (NEG); extraction cone, 4.0 V; source temperature, 120 °C (POS)/100 °C (NEG); desolvation temperature, 300 °C; and, desolvation gas (nitrogen), 600 L h^−1^. The high collision energy ramp ranged from 20 to 45 V.

A targeted analysis of eight specific catechins was performed using a Nexera X2 UHPLC system coupled to an LC-MS 8040 triple quadrupole mass spectrometer (Shimadzu, Kyoto, Japan). The UHPLC system was equipped with two pumps (LC-30AD), a system controller (CBM-20A), an autosampler (SIL-30AC), a degasser (DGU-20AS), and a column oven (CTO-20AC). The analytes were separated on an Acquity UPLC BEH C_18_ column (2.1 × 50 mm, 1.7 µm; Waters) at 30 °C using the same mobile phase as for the UHPLC-QTOF/MS method. The injection volume was 2 µL with an elution flow rate of 0.4 mL min.^−1^, and the optimized gradient condition was as follows: 0–10 min, 5–15% B; 10–10.2 min, 15–100% B; and, 10.2–11.5 min, 100% B. Separated analytes were ionized in ESI POS mode, and MS was performed in multiple reaction monitoring (MRM) mode. The pause time and dwell time were set as 3.0 and 5.0 ms, respectively. The precursor ion (*m*/*z*) and product ions (*m*/*z*) of each compound were as follows: EGC and GC (307.00 > 139.05, 151.05, 163.05), EC and C (291.05 > 123.15, 139.10, 165.10), EGCG and GCG (459.05 > 139.10, 151.10, 289.05), and ECG and CG (443.00 > 123.15, 139.10, 273.05). LabSolution LCMS software (version 5.89, Shimadzu) was used to process the acquired data.

The established method was validated for linearity, precision, and accuracy using a series of aqueous standard solutions of catechins. Since the catechin concentrations varied greatly among the samples, two linear ranges were used to establish regression curves for low concentration (0.25, 0.50, 1.0, 5.0, and 10 µg mL^−1^) and high concentration (10, 25, 50, 75, and 100 µg mL^−1^) ranges. Accordingly, the precision and accuracy were evaluated at low, middle, and high concentration points in both low and high concentration ranges. All of the analyses were repeated in triplicate.

### 3.5. Data Processing and Statistical Analysis

Waters MarkerLynx XS software was used for data peak detection, deconvolution, alignment, data reduction, and following normalization to total marker intensity. The datasets were eventually refined, such that they contained a list of mass and retention time pairs with corresponding intensities for all of the detected peaks from each data file. A total of 3099 and 478 variables were detected in the samples under POS and NEG modes, respectively. The main parameters for peak detection were set as follows: retention time range, 0.5–17 min.; mass range, 100–1200 Da; XIC window, 0.02 Da; mass window, 0.02 Da; and, retention time window, 0.10 min. After the peaks were recognized and aligned, the area of each peak was normalized to total marker intensity in each chromatogram. The parameters of peak integration were as follows: marker intensity threshold, 500 (NEG)/1000 (POS); peak width at 5% of the height, 1 s; peak-to-peak baseline noise, 0.00; without smoothing. The resulting data included retention time, *m*/*z* value, and normalized peak area that composed a dataset. The dataset was subjected to PCA and OPLS-DA without transformation using EZinfo 2.0 software (Waters, Milford, MA, USA). Statistical comparison in Figure 1, paired t-test and ANOVA, followed by Fisher’s LSD post hoc analysis, was conducted while using GraphPad Prism 6 for Windows (San Diego, CA, USA).

## 4. Conclusions

The present study presented the first comprehensive study on the effects of brewing conditions on green tea infusions. It showed that brewing temperature and time significantly affected metabolic profile changes, although the time effects were marginal at 60 °C. A total of 33 metabolites of various classes were identified as differential marker compounds that are associated with different brewing conditions, with some exhibiting drastically altered levels according to brewing temperature and/or time. An analysis of the metabolic profiles allowed the green tea infusions to be clearly classified into two clusters: infusions at 95 °C for ≥ 30 min and the others. Meanwhile, the infusions showed discernable difference in the antioxidant capacity between 60 °C and 95 °C, while the capacity change over time was generally mild. The highest levels of total catechins were observed at 60 °C for 60 min and at 95 °C for 10 min, after which they largely remained unaltered at similar values. However, the levels of the individual catechins changed in a different manner from the total catechins. The epi-structured catechins showed significant and continuous decline after 10 min at 95 °C, whereas they barely decreased at 60 °C after 60 min.

Collectively, the current study shows that the brewing conditions can differently affect the characteristics of green tea infusions in terms of the metabolic and catechin profiles and the antioxidant capacity. Therefore, it is suggested that simple interpretations of green tea infusions and extracts that are based on activity or total content of a group of compounds should be avoided. That is, the samples of green tea infusions should be carefully prepared in consideration of the effects of brewing conditions on the metabolic profiles and activity in the studies where green tea is subjected to chemical and biological investigation, including bioactivity assessment, origin discrimination, and quality assessment.

## Figures and Tables

**Table 1:** Identification results of differential metabolites in green tea infusions under different brewing conditions.

No.	t_R_ (min)	Compounds	Formula	Monoisotopic Molecular Mass	Detected Mass	Mass Error (mDa)
1	0.79	Glutamine ^1^	C_5_H_10_N_2_O_3_	146.0691	147.0761 ^a^	0.8
2	0.79	Glycerophosphocholine ^1^	C_8_H_20_NO_6_P	257.1028	258.1079 ^a^	2.7
3	0.8	Ribonic acid ^2^	C_5_H_10_O_6_	166.0477	165.0399 ^b^	0.0
4	0.8	Maltose ^1^	C_12_H_22_O_11_	342.1162	341.1081 ^b^	0.3
5	0.81	Quinic acid ^1^	C_7_H_12_O_6_	192.0634	191.0555 ^b^	0.1
6	0.83	Ascorbic acid ^2^	C_6_H_8_O_6_	176.0321	175.0241 ^b^	0.2
7	0.83	Glutathione ^2^	C_10_H_17_N_3_O_6_S	307.0838	308.0853 ^a^	6.3
8	0.84	Galloyl glucose isomer 1 ^2, d^	C_13_H_16_O_10_	332.0743	331.0660 ^b^	0.5
9	0.84	2-*O*-*p*-Coumaroylhydroxycitric acid ^2^	C_15_H_14_O_10_	354.0587	355.0668 ^a^	−0.3
10	1.15	Theanine ^1^	C_7_H_14_N_2_O_3_	174.1004	173.0922 ^b^	0.4
11	1.15	Cysteinylglycine ^2^	C_5_H_10_N_2_O_3_S	178.0412	179.0480 ^a^	1.0
12	1.15	Galloyl glucose isomer 2 ^2, d^	C_13_H_16_O_10_	332.0743	331.0663 ^b^	0.2
13	1.19	Pyroglutamic acid ^2^	C_5_H_7_NO_3_	129.0426	128.0350 ^b^	−0.2
14	1.19	Neuraminic acid ^2^	C_9_H_17_NO_8_	267.0954	268.1043 ^a^	−1.1
15	1.4	Galloyl glucose isomer 3 ^2, d^	C_13_H_16_O_10_	332.0743	331.0661 ^b^	0.4
16	1.64	Theogallin ^2^	C_14_H_16_O_10_	344.0743	345.0809 ^a^	1.2
17	1.69	Gallic acid ^1^	C_7_H_6_O_5_	170.0215	171.0285 ^a^; 169.0135 ^b^	0.8; 0.2
18	2.19	Vanillic acid ^2^	C_8_H_8_O_4_	168.0423	169.0497 ^a^	0.4
19	2.25	Gallocatechin ^1^	C_15_H_14_O_7_	306.0740	307.0813 ^a^; 305.0660 ^b^	0.5; 0.2
20	3.16	Epigallocatechin ^1^	C_15_H_14_O_7_	306.0740	307.0815 ^a^; 305.0663 ^b^	0.3; −0.1
21	3.44	*p*-Coumaroylquinic acid isomer 1 ^2, d^	C_16_H_18_O_8_	338.1002	361.0886 ^c^; 337.0925 ^b^	1.4; −0.1
22	3.78	Catechin ^1^	C_15_H_14_O_6_	290.0790	291.0863 ^a^; 289.0711 ^b^	0.5; 0.1
23	4.9	*p*-Coumaroylquinic acid isomer 2 ^2, d^	C_16_H_18_O_8_	338.1002	361.0897 ^c^; 337.0923 ^b^	0.3; 0.1
24	5.36	Epicatechin ^1^	C_15_H_14_O_6_	290.0790	291.0867 ^a^; 289.0712 ^b^	0.1; 0.0
25	5.57	*p*-Coumaroylquinic acid isomer 3 ^2, d^	C_16_H_18_O_8_	338.1002	361.0889 ^c^; 337.0922 ^b^	1.1; 0.2
26	5.66	Epigallocatechin gallate ^1^	C_22_H_18_O_11_	458.0849	459.0921 ^a^; 457.0766 ^b^	0.6; 0.5
27	6.43	Gallocatechin gallate ^1^	C_22_H_18_O_11_	458.0849	459.0920 ^a^; 457.0767 ^b^	0.7; 0.4
28	7.03	*p*-Coumaroylquinic acid isomer 4 ^2, d^	C_16_H_18_O_8_	338.1002	361.0905 ^c^; 337.0941 ^b^	−0.5; −1.7
29	7.33	Myricetin-3-*O*-glucoside ^2^	C_21_H_20_O_13_	480.0904	479.0814 ^b^	1.2
30	9.42	Epicatechin gallate ^1^	C_22_H_18_O_10_	442.0900	443.0972 ^a^; 441.0814 ^b^	0.6; 0.8
31	9.8	Catechin gallate ^1^	C_22_H_18_O_10_	442.0900	443.0977 ^a^; 441.0824 ^b^	0.1; −0.2
32	10.39	Kaempferol 7-galactoside 3-rutinoside ^2^	C_33_H_40_O_20_	756.2113	755.2025 ^b^	1.0
33	11.61	(−)-Epiafzelechin 3-gallate ^2^	C_22_H_18_O_9_	426.0951	425.0863 ^b^	1.0

^1^ Identified metabolite (confirmed using authentic standards). ^2^ Putatively annotated compound. ^a^ [M-H]^+^. ^b^ [M-H]^−^. ^c^ [M+Na]^+^. ^d^ See Supplementary Materials for putative identification.

## Supplementary Materials

The following are available online, Figure S1: Three-dimensional PCA score plots obtained from UHPLC-QTOF/MS analysis of green tea infusions brewed at 60 °C and 95 °C for 5–300 min. in positive mode (a) and negative mode (b), Figure S2: Relative intensity changes of green tea infusions brewed at 60 °C and 95 °C for 5–300 min, Figure S3: Representative MRM chromatograms of eight catechins at 10 μg mL^−1^ by UHPLC-QqQ/MS analysis in POS mode, Table S1: Linearity, LOD and LOQ of the developed UHPLC-QqQ/MS method, Table S2: Precision and accuracy results of the developed UHPLC-QqQ/MS method.
