# Supplementary material for: Comprehensive Investigation of the Effects of Brewing Conditions in Sample Preparation of Green Tea Infusions
Source: Molecules. 2019 May 4;24(9):1735. doi: 10.3390/molecules24091735 (PMC6539062; doi:10.3390/molecules24091735)
Supplement: Supplementary file 1 [file molecules-24-01735-s001.pdf]

Supplementary Information

Comprehensive investigation of the effects of brewing conditions in sample preparation of green tea infusions

Yan Jin[1], Jing Zhao[2], Eun Mi Kim[1], Ki Hyun Kin[1], Seulgi Kang[1], Heesoo Lee[1], Jeongmi Lee[1,*]

[1] School of Pharmacy, Sungkyunkwan University, Suwon, 16419, Republic of Korea

[2] School of Pharmacy, Shenyang Pharmaceutical University, Shenyang, Liaoning, 110016, China

[*]Corresponding author: jlee0610@skku.edu; jlee0610@gmail.com

Tel.: +82-31-290-7784/ Fax: +82-31-292-8800

Putative identification of differential marker compounds

In Table 1, four isomers of *p*-coumaroylquinic acid (*p*-CQA) and three isomers of galloyl glucose are included. Specifically, four peaks with retention times (t_R) of 3.44 min, 4.90 min, 5.57 min, and 7.03 min had the same m/z values of 361.08 ($[M+Na]^+$) in POS mode and 337.09 ($[M-H]^-$) in NEG mode. Putatively identified as *p*-CQA isomers, they could not be discretely identified only based on MS^n information and further differentiation was not pursued due to limited accessibility to the individual standard compounds. Similarly, three galloyl glucose isomers that eluted at 0.84 min, 1.15 min, and 1.40 min with the same m/z value of 331.07 ($[M-H]^-$) were not individually identified. Zhao et al. reported the presence of galloyl glucose and its quantification in green tea; however, they did not consider its isomeric differentiation [1].

Figure S1. Three-dimensional PCA score plots obtained from UHPLC-QTOF/MS analysis of green tea infusions brewed at 60 ˚C and 95 ˚C for 5-300 min in positive mode (a) and negative mode (b).

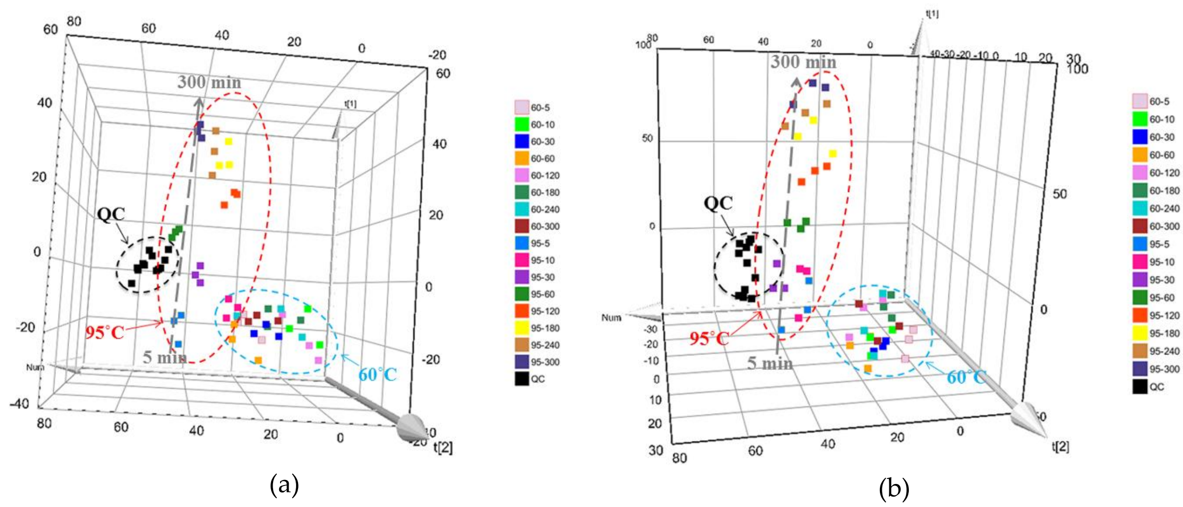

Figure S2. Relative intensity changes of green tea infusions brewed at 60˚C and 95˚C for 5-300 min. The dashed and solid lines indicate brewing at 60˚C and 95˚C, respectively. (a) *p*-CQA isomer 1, (b) *p*-CQA isomer 2, (c) *p*-CQA isomer 3, (d) *p*-CQA isomer 4, (e) galloyl glucose isomer 1, (f) galloyl glucose isomer 2, (g) galloyl glucose isomer 3, (h) gallic acid, (i) vanillic acid, (j) glutamine, (k) theanine, (l) pyroglutamic acid, (m) ribonic acid, (n) quinic acid, (o) neuraminic acid, and (p) kaempferol 7-galactoside 3-rutinoside.

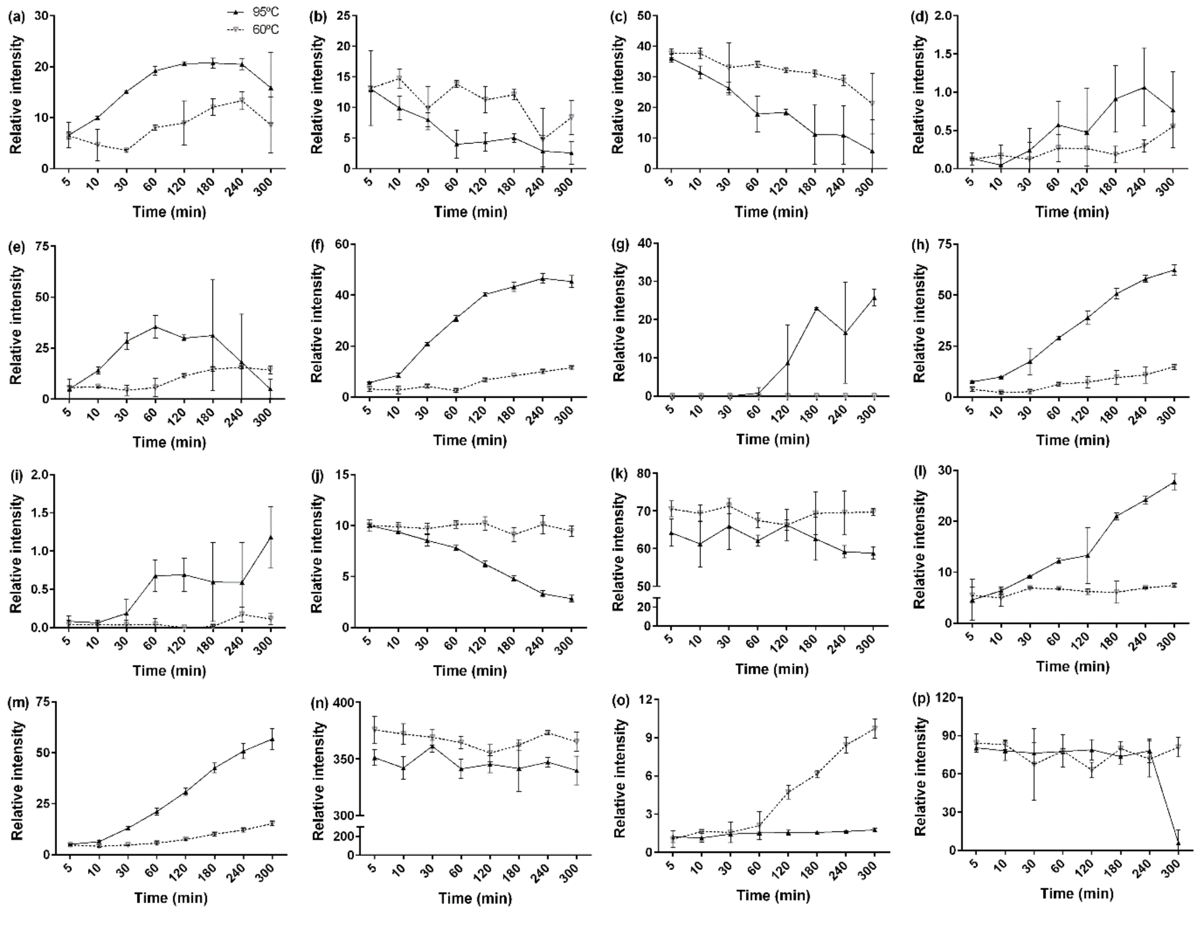

Figure S3. Representative MRM chromatograms of eight catechins at 10 μg mL^{-1} by UHPLC-QqQ/MS analysis in POS mode.

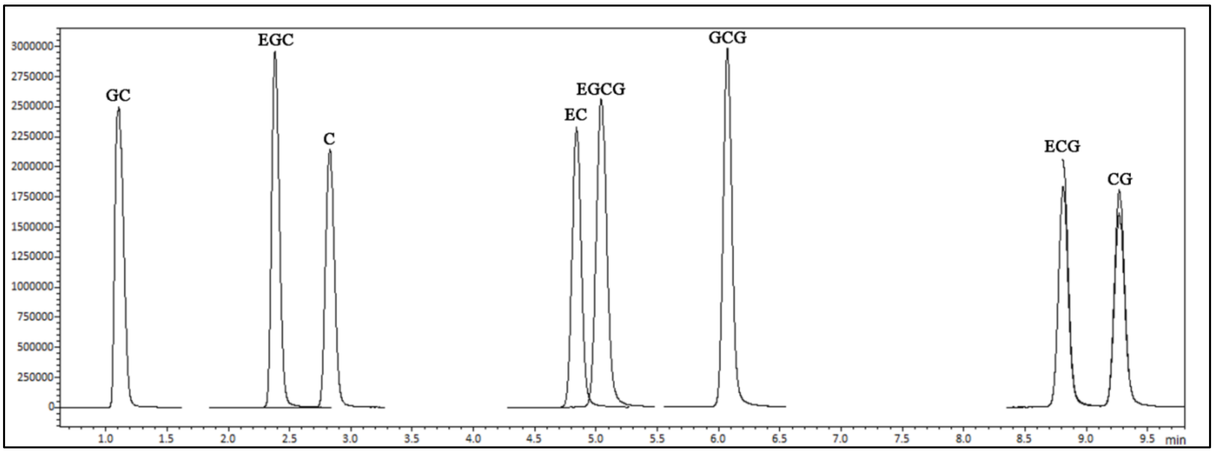

Table S1. Linearity, LOD and LOQ of the developed UHPLC-QqQ/MS method.

Analyte	Low concentration range (0.25-10 μg mL^{-1})				High concentration range (10-100 μg mL^{-1})	
	Calibration curve	r^2	LOD [a]	LOQ [b]	Calibration curve	r^2
GC	y = 1008000x - 63325	0.9996	0.010	0.030	y = 906131x + 1890000	0.9971
EGC	y = 1081000x - 96190	0.9997	0.001	0.003	y = 964347x + 2710000	0.9958
C	y = 642644x - 20300	0.9997	0.025	0.077	y = 562921x + 1451000	0.9965
EC	y = 678274x - 54113	0.9995	0.018	0.056	y = 597967x + 1473000	0.9953
EGCG	y = 1061000x - 79853	0.9996	0.005	0.016	y = 842489x+ 3542000	0.9955
GCG	y = 1023000x - 73512	0.9997	0.004	0.014	y = 816892x + 3112000	0.9959
ECG	y = 710516x - 26473	0.9995	0.019	0.058	y = 557543x + 2382000	0.9966
CG	y = 682118x - 31332	0.9990	0.025	0.077	y = 575746x + 1843000	0.9972

[a] μg mL^{-1}. Calculated from 3.3 times the SD of peak areas obtained by samples at 0.25 μg mL^{-1} ($\sigma_{0.25}$) divided by the slope (S_{low}) of the calibration curve of low concentration range.

[b] μg mL^{-1}. $10 \times \sigma_{0.25}/S_{\text{low}}$.

Table S2. Precision and accuracy results of the developed UHPLC-QqQ/MS method.

Analyte	Low concentration range (0.25-10 μg mL^{-1})			High concentration range (10-100 μg mL^{-1})		
	Concentration [a]	Precision (% RSD) [b]	Accuracy (%) [b]	Concentration [a]	Precision (RSD %) [b]	Accuracy (%) [b]
GC	0.25	1.36	112.4	10	1.55	89.8
	1.0	2.15	95.8	50	1.41	102.6
	10	1.55	100.1	100	2.62	98.36
EGC	0.25	1.24	118.2	10	1.15	83.0
	1.0	0.70	92.8	50	0.80	103.2
	10	1.15	100.0	100	1.90	97. 7
C	0.25	3.33	105.0	10	1.60	88.1
	1.0	1.63	98.5	50	1.90	102.9
	10	1.60	100.1	100	3.10	98.4
EC	0.25	2.59	118.1	10	1.42	88.4
	1.0	1.17	98.4	50	2.86	100.8
	10	1.42	100.5	100	1.55	97.4
EGCG	0.25	0.77	113.9	10	1.10	83.1
	1.0	3.58	95.5	50	3.74	103.1
	10	1.10	100.0	100	1.98	97.8

GCG	0.25	0.64	113.6	10	0.99	86.5
	1.00	4.67	97.7	50	1.84	101.8
	10.00	0.99	100.2	100	2.78	98.0
ECG	0.25	2.55	105.8	10	2.02	84.0
	1.00	3.40	96.5	50	0.73	102.9
	10.00	2.02	99.8	100	2.90	98.2
CG	0.25	3.48	107.1	10	2.76	85.5
	1.00	2.98	94.5	50	1.54	102.9
	10.00	2.76	99.7	100	2.96	98.8

[a] $\mu g\ mL^{-1}$.

[b] n=3.

References for SI

1. Zhao, Y.; Chen, P.; Lin, L.; Harnly, J.; Yu, L. L.; Li, Z., Tentative identification, quantitation, and principal component analysis of green pu-erh, green, and white teas using UPLC/DAD/MS. *Food Chem* **2011,** 126, (3), 1269-1277.
